# Two new species of the genus *Conocybe* (Agaricales, Bolbitiaceae) from Punjab, Pakistan

**DOI:** 10.3897/mycokeys.129.177354

**Published:** 2026-02-27

**Authors:** Yilan Jin, Muhammad Haqnawaz, Abdul Rehman Niazi, Pingzhu Lu, Abdul Nasir Khalid, Qirui Li

**Affiliations:** 1 Agricultural College, Anshun University, Anshun, Guizhou Province, 561000, China Anshun University Anshun China; 2 Fungal Biology and Systematics Research Laboratory, Institute of Botany, University of the Punjab, Quaid–e–Azam Campus 54590, Lahore, Pakistan University of the Punjab Lahore Pakistan; 3 Chongqing Three Gorges Medical College, Wanzhou Chongqing, 404100, China Guizhou Medical University Guiyang China; 4 The High Efficacy Application of Natural Medicinal Resources Engineering Centre of Guizhou Province (The Key Laboratory of Optimal Utilization of Natural Medicine Resources), School of Pharmaceutical Sciences, Guizhou Medical University, Guiyang, Guizhou, 550004, China Chongqing Three Gorges Medical College Wanzhou Chongqing China

**Keywords:** Basidiomycetes, macrofungal diversity, mushrooms, phylogenetic analysis, Pilosellae, taxonomy

## Abstract

In this study, two new species of genus *Conocybe* are described, based on morpho-anatomical and phylogenetic analyses of ITS and LSU (28S) regions. According to phylogenetic analyses, *Conocybe kotadduensis* & *C. safariensis* make a separate clade with a strong bootstrap value from the closest species of the genus *Conocybe*, sect. *Pilosellae*. *Conocybe kotadduensis* is closest to *C. nitrophila*, while *C. safariensis* is closer to *C. ceracea*. *Conocybe kotadduensis* differs from *C. nitrophila* due to pale yellow, parabolic pileus with velvety surface, scabrous surface of stipe, amygdaliform to ovoid, yellowish basidiospores, broad, fusiform, cylindrical cells of lecythiform cheilocystidia, sphero-pedunculate to obovoid, globose to sub-globose pileipellis elements. *Conocybe safariensis* can be distinguished from *C. ceracea* in having a conical to convex pileus, orange to light gray stipe, fusiform to lacrymoid basidiospores, obovoid to subglobose basidia, ellipsoid to globose cheilocystidia, and absence of pleurocystidia.

## Introduction

Genus *Conocybe* Fayod (Bolbitiaceae), is widely distributed in the world and mostly found in temperate regions of Asia, on herbivorous dung, fertile soil, grassland, and lawns etc. (Hausknecht 2009; Song and Bau 2023). It is a toxic fungus that contains toxic substances, such as phallotoxins, psilocybin, and amatoxins, which cause health problems like liver damage, gastroenteritis, and neuropsychological problems (Wu et al. 2019; Song and Bau 2023; Song and Bau 2025). However, these toxic substances also have shown significant pharmacological activities, such as treatment-resistant depression and post-traumatic stress disorder (Griffiths et al. 2016; Song and Bau 2023; Song and Bau 2025). It is characterized by a conical pileus, brown-rusty lamellae, and slender stipe often covered with powdery or hairy, lecythiform cheilocystidia (Song and Bau 2023; Song and Bau 2025). The genus *Conocybe* consist of 12 sections, viz., *Conocybe* sect. *Candidae* Watling, sect. *Conocybe* Fayod, sect. *Conocybella* (Singer) Watling, sect. *Giganteae* Singer, sect. *Heterocystidiae* E.F. Malysheva, sect. *Inopinatae* Hauskn. & Contu, sect. *Mixtae* Singer, sect. *Nodulososporae* Watling, sect. *Obscurae* Hauskn. & Krisai, sect. *Ochromarasmius* (Singer) Hauskn. & Krisai, sect. *Pilosellae* Singer, and sect. *Singerella* Watling (Hausknecht and Contu 2007; Hausknecht and Krisai-Greilhuber 2007; Malysheva 2017; Song and Bau 2023; Asif et al. 2025). Our two newly identified taxa belong to sect. *Pilosellae*, distinguished by an incomplete white stipe, numerous hairs, and non-lecythiform caulocystidia, with *C. pilosella* as the type species. From Pakistan, only 14 species have been previously reported (Akram et al. 2025; Asif et al. 2025). The aim of this work is to generalize the current knowledge about species diversity of this genus and to provide the detailed descriptions of the species collected from the Pakistan.

## Materials and methods

### Sampling and study area (Fig. 1)

Specimens were collected during Jun-November 2020–2023, in Bet Faqirwali and Safari Park, bed of the Indus River, District Kot Addu, (30°14'44"N, 70°51'04"E, 134 m a.s.l.), Punjab Province, Pakistan. The climate of Kot Addu is very hot during summer and mild in winter with an approximate highest and lowest temperature of 51 °C and -1 °C respectively; average annual rainfall is 127 mm (Haqnawaz et al. 2023a, 2024a, b; Li et al. 2025). Vegetation of the sampling site is dominated by subtropical plant species such as *Albizia chinensis* (Osbeck) Merrill *Calotropis procera* (Ait.) Ait., Hort., *Dalbergia sissoo* Roxb., *Mangifera indica* L. *Saccharum bengalense* Retz., *S. officinarum* L., *Tamarix aphylla* (L.) Warb., and *Vachellia nilotica* (L.) P.J.H.Hurter & Mabb, (Stewart 1972; Haqnawaz et al. 2023a, b). Samples were photographed, tagged, and then dried in front of the electric fan heater at about 35 °C temperature and were deposited in the Lahore Herbarium (LAH), Institute of Botany, University of the Punjab, Lahore, Pakistan.

**Figure 1.**
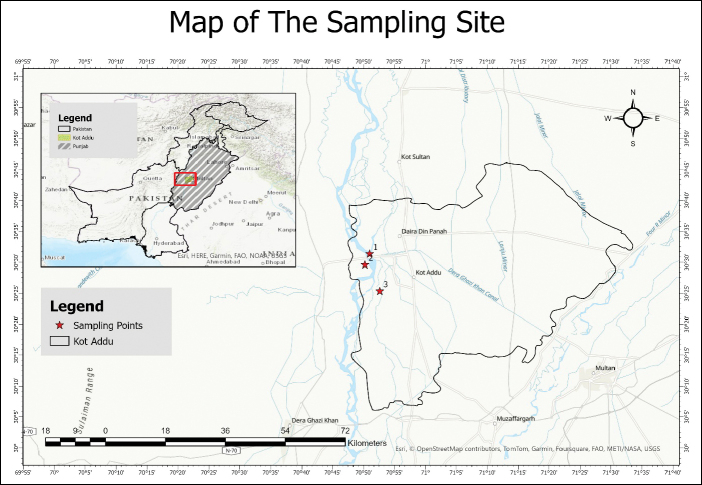
Maps of sampling sites where the novel taxa were found, shows with the red color.

### Morpho-anatomical characterization

Macroscopic characteristics such as size, shape, and color of pileus and stipe were noted, based on fresh samples. Description terminology by Vellinga (2001) was followed, while for color determination, the Munsell color chart was followed (Munsell 1994). Congo red was used for dyeing. The microscopic features like size, shape, and color of basidiospores, basidia, cheilocystidia, pileipellis, and caulocystidia were observed using a compound microscope (OLYMPUS BX43, Tokyo, Japan), and measurements were taken using calibrated Piximètre software connected to a compound microscope through a HDCE-X5 microscopic camera. In the case of basidiospores, at least 50 spores were measured. The formula [n/m/p] indicates ‘n’ the number of basidiospores measured from ‘m’ basidiomata of ‘p’ collection. Basidiospores measurements are presented as (a–) b–c (–d), where b–c indicates 90% of the calculated values, while values in parenthesis are extreme lowest or extreme highest values; Q indicates the individual spore length/width ratio while Qav. is the average length/width ratio of all basidiospores (Ge et al. 2010).

### Molecular phylogenetic analyses

DNA was extracted from the dried specimens using the 2% CTAB method (Bruns 1995). Primers used during amplifications were ITS1F 5’-CCT GGT CAT TTA GAG GAA GTA A-3’ as forward and ITS4 5’-TCC TCC GCT CTA TTG ATA TGC-3’ as reverse for nrITS region (>775 bp) while LROR 5’-ACC CGC TGA ACT TAA GC-3’ as forward and LR5 5’-TCC TGA GGG AAA CTT CG-3’ as reverse for nrLSU region (>910 bp) (Gardes and Bruns 1993, White et al. 1990). The Polymerase Chain Reaction (PCR) was performed in a 50 µl reaction volume: containing 5 µl 10× Econo Taq Buffer (Lucigen, Middleton, Wisconsin, USA), 1 µl dNTPs, 2.5 µl of each primer (10 µM/µl), 0.25 µl of Econo Taq® DNA Polymerase (Lucigen), 28.75 µl H_2_O and 10 µl DNA template. All PCR amplifications were carried out with the cycling program for 35 cycles as follows, denaturation at 94 °C for 30 sec, annealing at 54 °C for 1 min, extension at 71 °C for 2 min, with an initial denaturation at 94 °C for 2 min and a final extension of 71 °C for 5 min. PCR products were then sequenced and analyzed (Usman and Khalid 2020; Haqnawaz et al. 2023b).

**Figure 2.**
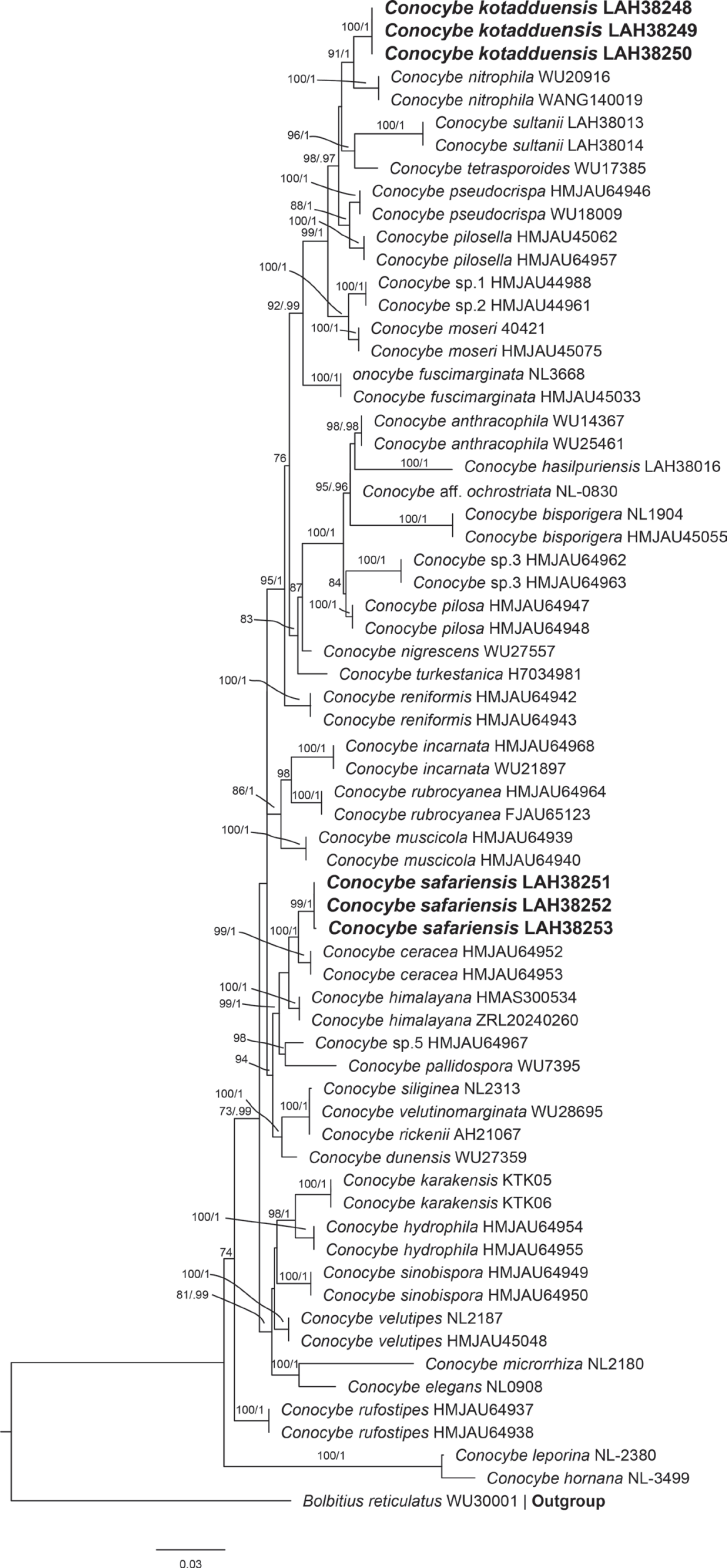
Phylogenetic tree of the genus *Conocybe* as generated by maximum likelihood (ML) and Bayesian analyses, based on combined ITS and LSU sequences. Bootstrap values ≥ 50%, based on 1,000 replicates and Bayesian posterior probabilities (PP) ≥ 0.90, are shown at the branches. Novel sequences, generated during this study, are shown in bold.

Forward and reverse primer reads of both ITS and nrLSU regions were combined and consensus sequences were obtained using Bioedit. ver. 7.2.5 (Hall 1999). The resulting sequences were subjected to a BLAST search at NCBI (https://www.ncbi.nlm.nih.gov/guide/). The sequences of closely related species from GenBank and sequences used from a recent publication of *Conocybe*, were included in our final phylogenetic tree (Asif et al. 2025; Song and Bau 2025). One sequence of *Bolbitius reticulatus* (Pers.) Ricken was used as an outgroup for the ITS and LSU datasets. For the phylogenetic analyses, a Clustal W MUSCLE alignment was implemented in BioEdit v. 7.2.5 with manual adjustments. A combined (ITS–LSU) maximum likelihood phylogenetic tree was constructed using RAxML-HPC2 v. 8.2.12 on XSEDE (8.2.10) implemented on the CIPRES Science Gateway (Miller et al. 2010). The GTR+GAMMA nucleotide substitution model was used, with 1,000 bootstrap iterations performed using rapid bootstrapping. The resulting bootstrap values were then mapped on to the best-scoring ML tree and show in Fig. 2. The model of evolution was estimated by MrModeltest 2.2 (Nylander 2004). Markov chain Monte Carlo (MCMC) sampling in MrBayes v. 3.2.2 (Ronquist et al. 2012) was used to determine posterior probabilities (PP). Six simultaneous Markov chains were run for 1,000,000 generations, and trees were sampled every 1,000^th^ generation. Bootstrap values ≥ 50% and Bayesian PP ≥ 0.90 are indicated on the branches, which were visualized in FigTree v. 1.4.2 (Rambaut 2014). The newly generated sequences were submitted to GenBank, and short descriptions of the species were deposited in MycoBank (Robert et al. 2013). Boot strap values ≥ 50% are mentioned on the branches that were visualized in FigTree v. 1.4.2 (Rambaut 2014). The newly generated sequences were deposited in GenBank, and short descriptions of the species were deposited in MycoBank (Robert et al. 2013). All the sequences used in the final phylogenetic tree are presented in Table 1, together with voucher numbers, GenBank accession numbers, and country of origin. The newly generated sequences were deposited in GenBank and are in bold in the phylogenetic trees and Table 1.

**Table 1.** Fungal species used for phylogenetic analyses of *Conocybe* including their species names, locality, voucher and GenBank accession numbers of the nrITS & nrLSU regions. Sequences generated for this study are shown in bold.

Species names	Locality	Voucher	GenBank No.	
			ITS	LSU
*Conocybe anthracophila*	Italy	WU25461	JX968237	JX968355
*Conocybe anthracophila*	Italy	WU14367	JX968212	JX968329
*Conocybe aff. Ochrostriata*	Hungary	NL-0830	JX968236	JX968354
*Conocybe bisporigera*	China	HMJAU45055	OP526418	-
*Conocybe bisporigera*	Hungary	NL-1904	JX968235	JX968353
*Conocybe ceracea*	China	HMJAU64952	OQ758111	OQ758219
*Conocybe ceracea*	China	HMJAU64953	OQ758112	OQ758220
*Conocybe dunensis*	Spain	WU27359	JX968227	JX968345
*Conocybe elegans*	Sweden	NL-0908	JX968223	JX968341
*Conocybe fuscimarginata*	Sweden	NL-3668	JX968238	JX968356
*Conocybe fuscimarginata*	China	HMJAU45033	OQ780310	OQ758208
*Conocybe hasilpuriensis*	Pakistan	LAH38016	PP817194	OR773176
*Conocybe hornana*	Slovakia	NL-3499	JX968178	JX968294
*Conocybe himalayana*	China	HMAS 300534	PQ099842	PP968799
*Conocybe himalayana*	China	ZRL20240260	PQ699279	PQ699303
*Conocybe hydrophila*	China	HMJAU64954	OQ758116	OQ758232
*Conocybe hydrophila*	China	HMJAU64955	OQ758117	OQ758233
*Conocybe incarnate*	China	HMJAU64968	OQ780316	-
*Conocybe incarnate*	Finland	WU21897	JX968229	JX968347
*Conocybe juniana*	Sweden	NL-2105	JX968191	JX968307
*Conocybe karakensis*	Pakistan	KTK05	ON392730	-
*Conocybe karakensis*	Pakistan	KTK06	ON392731	
*Conocybe kotadduensis*	Pakistan	LAH38248	PQ409233	PQ409236
*Conocybe kotadduensis*	Pakistan	LAH38249	PQ409234	PQ409237
*Conocybe kotadduensis*	Pakistan	LAH38250	PQ409235	PQ409238
*Conocybe leporina*	Hungary	NL-2380	JX968177	JX968293
*Conocybe microrrhiza*	Hungary	NL-2180	JX968222	JX968340
*Conocybe moseri*	Germany	40421	MK412354	-
*Conocybe moseri*	China	HMJAU45075	OQ780309	OQ758207
*Conocybe muscicola*	China	HMJAU64939	OQ758113	OQ758223
*Conocybe muscicola*	China	HMJAU64940	OQ758114	OQ758224
*Conocybe reniformis*	China	HMJAU64942	OQ758108	OQ758229
*Conocybe reniformis*	China	HMJAU64943	OQ758109	OQ758228
*Conocybe nigrescens*	Italy	WU27557	JX968234	JX968352
*Conocybe nitrophila*	India	WU20916	JX968233	JX968351
*Conocybe nitrophila*	China	WANG140019	KR998384	-
*Conocybe pallidospora*	Austria	WU7395	JX968239	JX968357
*Conocybe pilosa*	China	HMJAU64947	OQ758122	OQ758222
*Conocybe pilosa*	China	HMJAU64948	OQ758123	OQ758221
*Conocybe pilosella*	China	HMJAU45062	OQ780305	OQ758205
*Conocybe pilosella*	China	HMJAU64957	OQ780306	OQ758206
*Conocybe pseudocrispa*	China	HMJAU64946	OQ780307	OQ758212
*Conocybe pseudocrispa*	Austria	WU18009	JX968230	JX968348
*Conocybe rickenii*	Spain	AH21067	MF142238	-
*Conocybe rubrocyanea*	China	FJAU65123	PP501388	PP501398
*Conocybe rubrocyanea*	China	HMJAU64964	PP501389	PP501399
*Conocybe rufostipes*	China	HMJAU64937	OQ758120	OQ758227
*Conocybe rufostipes*	China	HMJAU64938	OQ758121	OQ758226
*Conocybe safariensis*	Pakistan	LAH38251	PP973138	PP973144
*Conocybe safariensis*	Pakistan	LAH38252	PP973139	PP973145
*Conocybe safariensis*	Pakistan	LAH38253	PP973140	-
*Conocybe siliginea*	Swedan	NL-2313	JX968225	JX968343
*Conocybe sinobispora*	China	HMJAU64949	OQ758118	OQ758230
*Conocybe sinobispora*	China	HMJAU64950	OQ758119	OQ758231
*Conocybe* sp. 1	China	HMJAU44988	OQ749737	OQ740305
*Conocybe* sp. 2	China	HMJAU44961	OQ749738	-
*Conocybe* sp. 3	China	HMJAU64962	OQ749739	OQ740306
*Conocybe* sp. 4	China	HMJAU64963	OQ749740	OQ740307
*Conocybe* sp. 5	China	HMJAU64967	OQ749741	-
*Conocybe sultanii*	Pakistan	LAH38013	OR773181	OR773183
*Conocybe sultanii*	Pakistan	LAH38014	OR773180	OR773182
*Conocybe turkestanica*	Turkestan	H7034981	H7034981	MH055382
*Conocybe tetrasporoides*	New Zealand	WU17385	JX968232	JX968350
*Conocybe velutinomarginata*	Germany	WU28695	JX968226	JX968344
*Conocybe velutipes*	Hungary	NL-2187	JX968228	JX968346
*Conocybe velutipes*	China	HMJAU45048	OQ780311	OQ758209
*Bolbitius reticulatus Outgroup*	Hungary	WU30001	JX968249	JX968366

## Results

### ITS & LSU phylogenetic analyses (Fig. 2, Table 1)

The combined phylogenetic tree of the nrITS & nrLSU region based on consists of 65 sequences, and *Bolbitius reticulatus* (WU30001) is an outgroup. Both new taxa are shown in bold, and formed separate clades from their closely related species. *Conocybe kotadduensis* formed a separate branch with 78% bootstrap support from its sister species, *Conocybe nitrophila* (Hauskn.) Yen W. Wang & S.S. Tzean (WU20916 & WANG140019); and the other closest species is *Conocybe pilosella* (Pers.) Kühner (HMJAU45062 & HMJAU64957). The second new species *Conocybe safariensis* also formed a separate branch from its sister species *C. ceracea* T. Bau & H.B. Song (HMJAU64952 & HMJAU64953) with high bootstrap value 94%, and *C. himalayana* Ke Wang, T.Z. Wei & P. Hong (HMAS300534 & ZRL20240260) with 89% bootstrap value.

### Taxonomy

#### Conocybe kotadduensis

Taxon classificationFungiAgaricalesBolbitiaceae

Haqnawaz, Niazi & Khalid sp. nov.

82AB4563-07F0-578D-8DB2-BF0990F2C48D

855941

Figs 3, 4

##### Etymology.

Species name “kotadduensis” (Latin) refers to the type locality Kot Addu, Punjab, Pakistan.

##### Holotype.

Pakistan • Punjab, Kot Addu District, bed of Indus River, (30°23'27"N, 70°48'28"E, 127 m a.s.l.), on plant debris, June 14, 2023, *Muhammad. Haqnawaz*, KA-06 (LAH38248). GenBank: PQ409233 (nrITS), PQ409236 (nrLSU).

##### Diagnosis.

*Conocybe kotadduensis* is different from the closest species, *C. nitrophila*, by its relatively light gray to pale yellow, parabolic absence of thick glutinous veil and viscid in regular to slightly crenate margins of pileus, adnexed, broad, thick lamellae with 2–3 tires of lamellulae, scabrous surface of stipe, amygdaliform to ovoid basidiospores, mostly broad bi-sterigmate of basidia, clavate, utriform with median constriction, fusiform, cylindrical shape of lecythiform cheilocystidia, sphero-pedunculate to obovoid, globose to sub-globose pileipellis elements.

**Figure 3.**
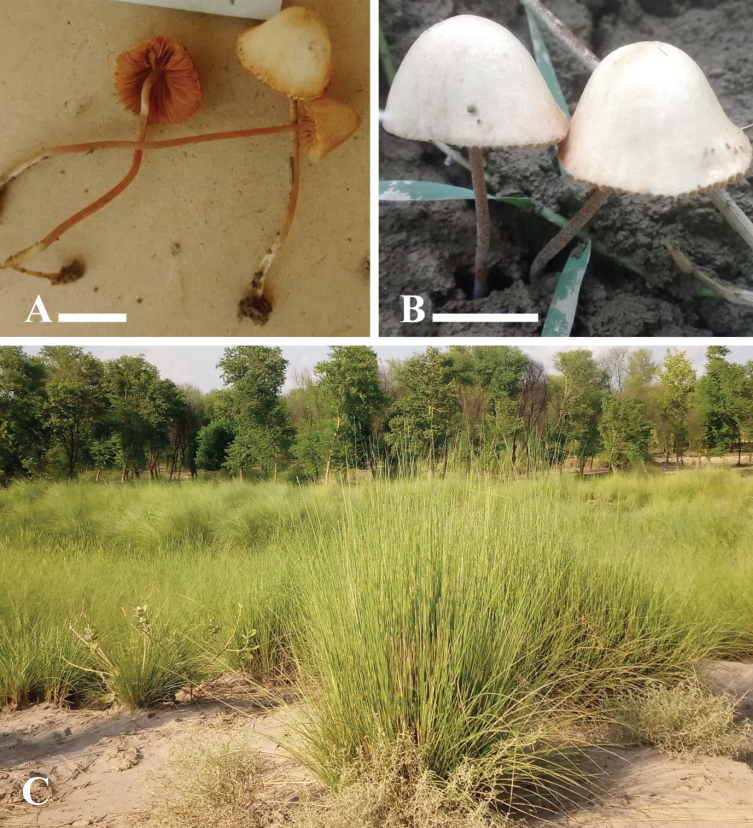
**A, B**. Basidiomata of *Conocybe kotadduensis*. (**A**. Holotype LAH38248); **C**. Sampling site. Scale bars: 10 mm (**A, B**).

##### Description.

***Pileus*** 10–13 mm diam., hemispherical to parabolic at young stage, conical, parabolic when old, straight shape of margins, regular to slightly crenate cap margins, velutinous surface, light gray (7.5 YR 8/1) to pale yellow (2.5 Y 8/4), with dull yellow orange (10YR 7/4) center. ***Lamellae*** adnexed, broad, thick, subdistant, fimbriate, unequal, with exceeding 1–3 tires, dull yellow orange (10 YR 7/4) to orange (7.5 YR 7/6). ***Stipe*** 25–49 mm in length, cylindrical, flexuous, central, with slightly scabrous surface, hard and dry, slightly broad toward base, orange (5 Y.R 6/6) at upper side, dull yellow orange (10 YR 7/4) at base, grayish white (N 8/0).

**Figure 4.**
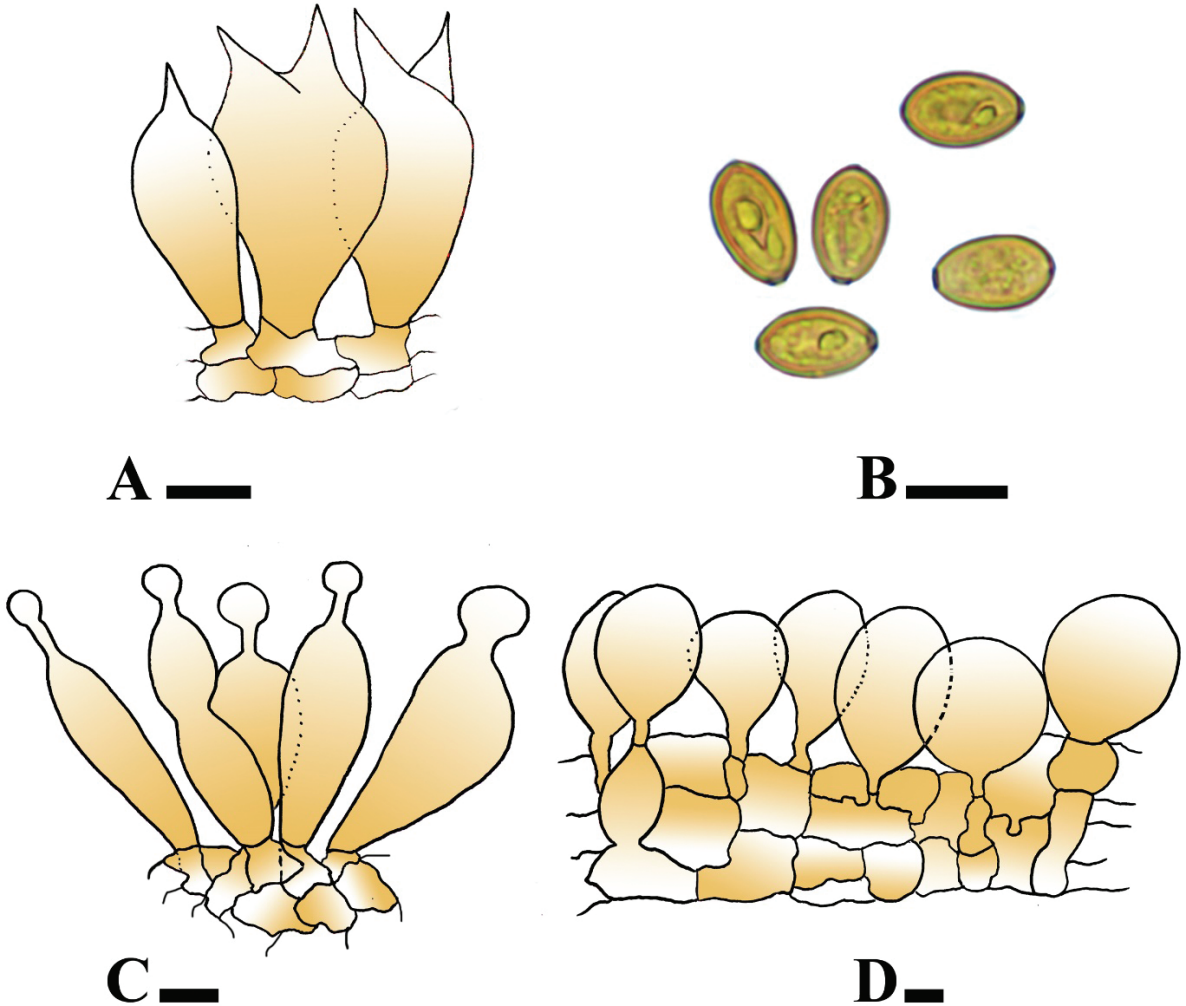
**A–D**. Microscopic structures of *Conocybe kotadduensis*. (**A**. Holotype LAH38248). **A**. Basidia; **B**. Basidiospores; **C**. Cheilocystidia; **D**. Pileipellis elements. Scale bars: 10 µm.

***Basidiospores*** [100/2/3], (13.2–)13.5–15.5 (–16) × (8–)8.5–9.5(–10) µm, avl × avw = 14.7 × 9.0 µm, Q = 1.47–1.69, Q_av_ = 1.62, amygdaliform to ovoid, yellowish to slightly light brown in 5% KOH, thin walled, guttulate, prominent germ pore. ***Basidia*** [30/2/3] (24–)24–28(–29) × (10.5–)11–11(–11.5) µm, clavate, with 2 sterigmata with broad width, hyaline in 5% KOH, thin-walled. ***Cheilocystidia*** [30/3/3] (14–) 14.5–24(–24.5) × (5.5–)6.–10.5(–11) µm, lecythiform with clavate, utriform with median constriction, fusiform, cylindrical shap of cells, rarely lageniform, hyaline, thin walled. ***Pileipellis*** [30/2/3] (25–89 × 1637 µm, sphero-pedunculate to obovoid, globose to sub-globose, hyaline and thin walled. ***Stipitipellis*** 8–23 µm, avw = 9.8 µm, cutis, hyaline in 5% KOH, thin walled, septate, unbranched, regular. ***Caulocystidia*** [15/3/3] 9–14 × 5–7 µm, rarely present, clavate to cylindrical, hyaline in 5% KOH, thin walled. Clamp connection absent.

##### Ecology and habitat.

Gregarious, terrestrial, under plants of *Saccharum bengalense* and *Cyperus rotundus*.

##### Additional specimens examined.

Pakistan • Punjab, Kot Addu, bed of Indus River, 30°25'19"N, 70°52'44"E, 132 m a.s.l., June 26, 2020, FW-98, *Muhammad Haqnawaz*, (***Paratype*** LAH38249), GenBank: PQ409234 [nrITS], PQ409237 [nrLSU]. • 30°14'44"N, 70°51'04"E, 134 m a.s.l. under *Cyperus rotundus*, August 15, 2021, TA-51, Muhammad Haqnawaz (***Paratype*** LAH38250), GenBank: PQ409235 (nrITS), PQ409238 (nrLSU).

#### Conocybe safariensis

Taxon classificationFungiAgaricalesBolbitiaceae

Niazi, Haqnawaz & Khalid sp. nov.

8478AAFB-264F-5B88-90FE-896C85021484

854578

Figs 5, 6

##### Etymology.

Species name safariensis (Latin) refers to the locality of the taxon. i.e., Safari Park, Kot Addu, Punjab, Pakistan.

##### Holotype.

Pakistan • Punjab, Kot Addu District, bed of Indus River, (30°31'33.63"N, 70°56'29.71"E, 135 m a.s.l.), on plant debris under *Tamarix aphylla*, July 05, 2021, *Muhammad. Haqnawaz*, HQ-69 (LAH38252). GenBank: PP973139 (nrITS), PP973145 (nrLSU).

##### Diagnosis.

*Conocybe safariensis* is different from the closest species, *C. ceracea*, by its hemispherical, lentiform, obtusely conical, then convex to plane and straight margin of pileus, orange to light gray stipe, obovoid to lacrymoid basidiospores, obovoid, ellipsoid to globose cheilocystidia, oblong, globose, clavate and fusiform elements of pileipellis, ellipsoid to broadly ellipsoid, clavate to broadly clavate caulocystidia, and absence of pleurocystidia.

**Figure 5.**
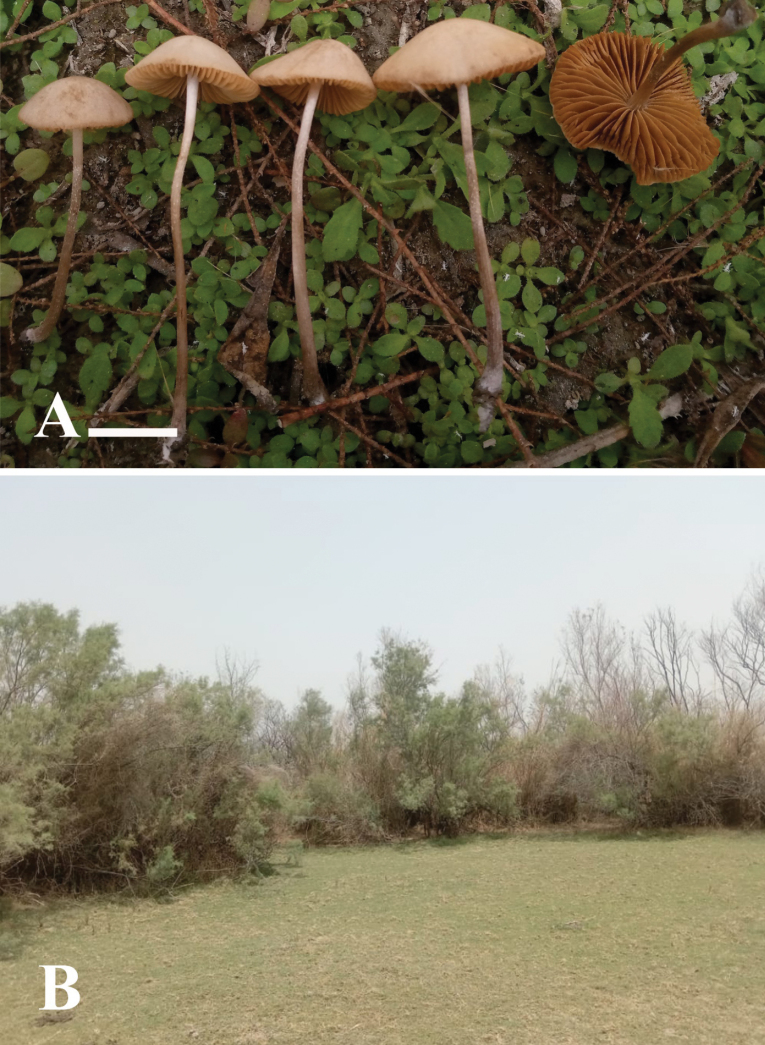
**A**. Basidiomata of *Conocybe safariensis*. (**A**. Holotype LAH38252); **B**. Sampling site, Scale bar: 10 mm (**A**).

##### Description.

***Pileus*** 15–24 mm long, hemispherical to campanulate at young stage, sub hemispherical, lentiform, obtusely conical, then convex to plane when old, slightly regular, straight shape and sulcate cap margins, smooth, light gray to dull orange (7.5 YR 8/2, 7/4), texture soft, thin, light gray (7.5 YR 8/2), ***Lamellae*** adnate, broad, average, sub distant, even, unequal, exceeding, and 3–7 tires, orange (7.5 YR 7/6). ***Stipe*** 25–49 mm in length, flexuous, white scabrous surface, costate surface, bulbous base, orange (7.5 YR 4/3) at the base, light brownish gray (7.5 YR 7/2) to light gray (5 YR 8/1) toward base.

**Figure 6.**
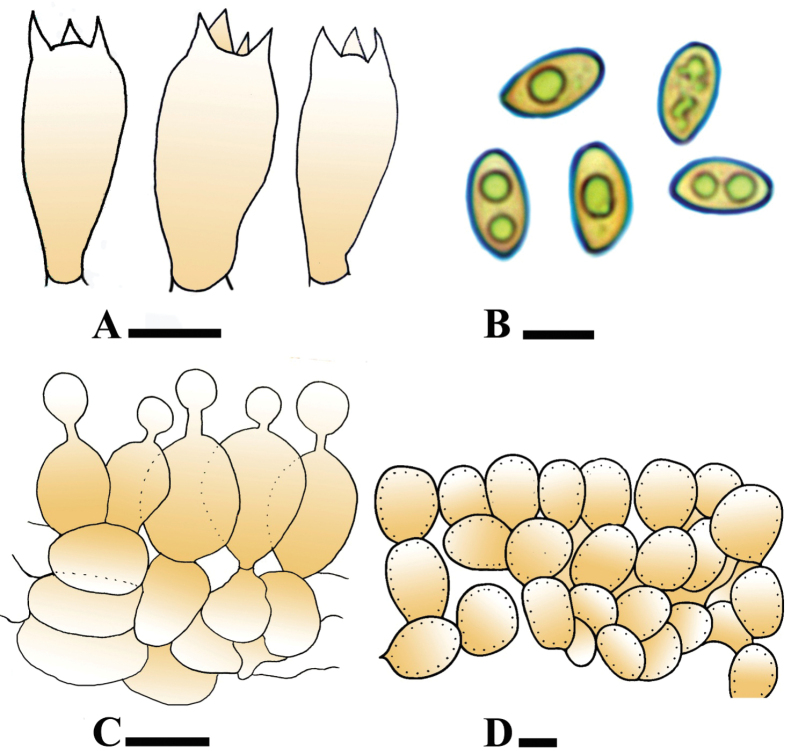
**A–D**. Microscopic structures of *Conocybe safariensis*. (**A**. Holotype LAH38252). **A**. Basidia; **B**. Basidiospores; **C**. Cheilocystidia; **D**. Pileipellis. Scale bars: 10 µm (**A–D**).

***Basidiospores*** [100/2/3], (8–) 8.6–10 (–10.5) × (4.5–) 4.8–5.5(–6) µm, avl × avw = 9.5 × 5.2 µm, Q = 1.63–2.2, Qav = 1.8, obovoid to lacrymoid, yellowish green to light reddish brown in water, guttulate, thick walled, prominent germ pore present. ***Basidia*** [30/2/3] (15–)16–23(–24) × (7–)8–12(–13) µm, clavate to broadly clavate, slightly cylindrical, with 1–4 sterigmata, hyaline in 5% KOH, amyloid, thin–walled, guttulate. ***Cheilocystidia*** [40/2/3] 16–25 × 8–12 µm, lecythiform with ellipsoid to broadly ellipsoid, globose to subglobose, clavate to broadly clavate cells, hyaline, thick walled. ***Pleurocystidia*** absent. ***Pileipellis*** [30/2/3] 20–50 × 18–36 µm, irregular epithelium, oblong, globose to subglobose, clavate to broadly clavate, fusiform elements, thin-walled. ***Stipitipellis*** 8–17 µm, avw = 12.5 µm, regular, trichoderm, hyaline, thin walled, septate, unbranched, regular, clamp connection present. ***Caulocystidia*** [30/2/3] 11–33 × 7–13 µm, rearly present, cylindrical ellipsoid to broadly ellipsoid, globose to subglobose, clavate to broadly clavate cells hyaline in 5% KOH.

##### Ecology and habitat.

Gregarious, terrestrial, under *Tamarix aphylla*, on loamy soil rich in organic matter.

##### Additional specimens examined.

Pakistan • Punjab, Kot Addu, Noor Shah Thal, 30°31'33.63"N, 70°56'29.71"E, 135 m a.s.l., September 29, 2022, *Muhammad Haqnawaz*, (***Paratype*** LAH38251), GenBank: PP973138 [nrITS], PP973144 [nrLSU]. • 30°25'21"N, 70°52'46"E 132 m. a.s.l., September 10, 2023, MQ-151, Muhammad Haqnawaz (***Paratype*** LAH38253), GenBank: PP973140 (nrITS).

## Discussion

Two new species, *Conocybe kotadduensis* and *C. safariensis*, were described based on phylogenetic analyses and morpho-anatomical descriptions. Phylogenetic analysis of nrITS and nrLSU sequence data showed that *Conocybe kotadduensis* and *C. safariensis* belong to section *Pilosellae* Singer (Song and Bau 2023). *Conocybe kotadduensis*, formed a separate branch from *C. nitrophila*, *C. pilosella*, and *C. pseudocrispa* with 18, 25, and 21 base pairs differences, respectively. While *Conocybe safariensis* is different from its sister’s species, *C. ceracea*, *C. himalayana* and *C. pallidospora* with 14, 19, and 38 base pairs differences, respectively.

Morpho-anatomically, *Conocybe kotadduensis* is distinguished from its sister species, *C. nitrophila*, by having pale yellow, parabolic pileus with velvety surface (vs. orange white, hygrophanous, hemispherical to a little convex pileus with smooth surface), scabrous surface of stipe (vs. smooth), amygdaliform to ovoid, yellowish to slightly light brown basidiospores (vs. broadly ellipsoid to oval, brownish orange to reddish brown), broad, fusiform, cylindrical cells of lecythiform cheilocystidia (vs. only clavate shape of cells), sphero-pedunculate to obovoid, globose to sub-globose pileipellis elements (vs. only broadly cylindrical to clavate). *C. nitrophila* has thick glutinous veil and viscid in fresh sample while absence in new taxon (Wang and Tzean 2015). The second closer species, *Conocybe pilosella* differs in having subpilose pileus covered with hair, tetra-sterigmate basidia, absent germpore, lecythiform pileocystidia (Song and Bau 2023). Another species, *C. pseudocrispa*, is different due to its oyster white surface with green beige to ivory center of pileus, narrowly adnate, beige-brown to ochre-brown lamellae, longitudinally striated, fine hairs, thick base of stipe, honey yellow to ochre-brown, ellipsoid to oblong basidiospores, subglobose, lageniform, lanceolate stipitipellis elements (Song and Bau 2023).

Similarly, morpho-anatomically, *Conocybe ceracea* differs from our second new taxon, *C. safariensis*, possessing subglobose, paraboloid to subcylindrical pileus, incurved margins, and presence of precipitate wax crystals on the surface of the pileus, honey yellow to melon yellow, elliptical to oblong and ovoid basidiospores, presence of pleurocystidia and subglobose, lageniform, cylindrical, or lanceolate caulocystidia (Song and Bau 2023). Similarly, *C. himalayana* a Chinese taxon, differs due to its decurved margins, subglobose, orange brown pileus with faintly pubescent surface, sometimes stipe forming a bulb, ovoid to ellipsoid basidiospores, lageniform cheilocystidia (Wang et al. 2024). Ecologically, *C. safariensis* is, terrestrial, grows under *Tamarix aphylla*, whereas its sister’s species *C. ceracea* grows under potted Orchidaceae plants, and *C. himalayana* grows on grassland (Song and Bau 2023; Wang et al. 2024). With these additions, the taxa of *Conocybe* known from Pakistan turn out to be 16.

## Supplementary Material

XML Treatment for Conocybe kotadduensis

XML Treatment for Conocybe safariensis

## Additional information

### Use of AI

No use of AI was reported.

### Author ORCIDs

Muhammad Haqnawaz https://orcid.org/0000-0003-4667-242X

Abdul Rehman Niazi https://orcid.org/0000-0002-1118-1148

Abdul Nasir Khalid https://orcid.org/0000-0002-5635-8031

Qirui Li https://orcid.org/0000-0001-8735-2890

### Data availability

All of the data that support the findings of this study are available in the main text or Supplementary Information.

## Supplementary materials

Supplementary material 1 ITS sequences Data type docx https://binary.pensoft.net/file/1550487This dataset is made available under the Open Database License (http://opendatacommons.org/licenses/odbl/1.0/). The Open Database License (ODbL) is a license agreement intended to allow users to freely share, modify, and use this Dataset while maintaining this same freedom for others, provided that the original source and author(s) are credited. Yilan Jin, Muhammad Haqnawaz, Abdul Rehman Niazi, Pingzhu Lu, Abdul Nasir Khalid, Qirui Li

Supplementary material 2 LSU sequences Data type fas https://binary.pensoft.net/file/1550488This dataset is made available under the Open Database License (http://opendatacommons.org/licenses/odbl/1.0/). The Open Database License (ODbL) is a license agreement intended to allow users to freely share, modify, and use this Dataset while maintaining this same freedom for others, provided that the original source and author(s) are credited. Yilan Jin, Muhammad Haqnawaz, Abdul Rehman Niazi, Pingzhu Lu, Abdul Nasir Khalid, Qirui Li
