# Supplementary material for: Two new species of the genus Conocybe (Agaricales, Bolbitiaceae) from Punjab, Pakistan
Source: MycoKeys. 2026 Feb 27;129:129–43. doi: 10.3897/mycokeys.129.177354 (PMC12966846; doi:10.3897/mycokeys.129.177354)
Supplement: Supplementary material 1 — ITS sequences [file mycokeys-129-129-s001.docx]

>LAH38248_*Conocybe_kotadduensis*

TTGAGGAAGTAAAAGTCGTAACAARKTYKCYGYRKRKKRACCTGCGGARGGATCATTACAGAGTAAACTTGGTGGGTTGTTGCTGGCTCTATACTGAGCATGTGCACACCTGTCATCTTTATCTTTCCACCATGTGCACCTTTTGTAGGACTGGAATAGACAATAAGCCTAGTCTCTTGGTAGACTATTGGAAAGTTGCTGATTTTATCAGCTCTTTCTGGTTCTGGCTGTCWATTTCATTCTCTACGATATTTATATACACACCATGTATGTTAATGAACGCAATCAATGGGCCCATAAAGCCTATAAACTATAATACAACTTTCAGCAACGGATCTCTTGGCTCTCGCATCGATGAAGAACGCAGCGAAATGCGATAAGTAATGTGAATTGCAGAATTCAGTGAATCATCGAATCTTTGAACGCACCTTGCGCTCCTTGGTATTCCGAGGAGCATGCCTGTTTGAGTGTCATTAAATTCTCAATCTTTACAACTTTGTGTTGTTCAGATTGGATTTGGGGGTTTTACTGTGAGGAATTCAATTCCTTCACTCCTCTTAAATGCATTAGCTGGAATGCCTCTGCATGTCTGACTATCAGTGTGATAATTATCTATACTGTAGTTGTTGCATGAGGGTATCTGCTTCCTCAATCGTCGCAAGACAAATGACCATCTTGACCTCAAATCAGGTAGRACYACYYGCTGAACTTAAGCATATCAAAA

>LAH38249_*Conocybe_kotadduensis*

TTGAGGAAGTAAAAGTCGTAAMAWKSKWTCCGTASGTGAACCTGCGGARGGATCATTACAGAGTAAACTTGGTGGGTTGTTGCTGGCTCTATACTGAGCATGTGCACACCTGTCATCTTTATCTTTCCACCATGTGCACCTTTTGTAGGACTGGAATAGACAATAAGCCTAGTCTCTTGGTAGACTATTGGAAAGTTGCTGATTTTATCAGCTCTTTCTGGTTCTGGCTGTCWATTTCATTCTCTACGATATTTATATACACACCATGTATGTTAATGAACGCAATCAATGGGCCCATAAAGCCTATAAACTATAATACAACTTTCAGCAACGGATCTCTTGGCTCTCGCATCGATGAAGAACGCAGCGAAATGCGATAAGTAATGTGAATTGCAGAATTCAGTGAATCATCGAATCTTTGAACGCACCTTGCGCTCCTTGGTATTCCGAGGAGCATGCCTGTTTGAGTGTCATTAAATTCTCAATCTTTACAACTTTGTGTTGTTCAGATTGGATTTGGGGGTTTTAYTGTGAGGAATTCAATTCCTTCACTCCTCTTAAATGCATTAGCTGGAATGCCTCTGCATGTCTGACTATCAGTGTGATAATTATCTATACTGTAGTTGTTGCATGAGGGTATCTGCTTCCTCAATCGTCGCAAGACAAATGACCATCTTGACCTCAAATCAGGTAGRMSYAYCCGCTGAACTTAAGCATATCAAAA

>LAH38250_*Conocybe_kotadduensis*

TTGAGGAAGTAAAAGTCGTAACAARKTYKCYGYRKRKKRACCTGCGGARGGATCATTACAGAGTAAACTTGGTGGGTTGTTGCTGGCTCTATACTGAGCATGTGCACACCTGTCATCTTTATCTTTCCACCATGTGCACCTTTTGTAGGACTGGAATAGACAATAAGCCTAGTCTCTTGGTAGACTATTGGAAAGTTGCTGATTTTATCAGCTCTTTCTGGTTCTGGCTGTCWATTTCATTCTCTACGATATTTATATACACACCATGTATGTTAATGAACGCAATCAATGGGCCCATAAAGCCTATAAACTATAATACAACTTTCAGCAACGGATCTCTTGGCTCTCGCATCGATGAAGAACGCAGCGAAATGCGATAAGTAATGTGAATTGCAGAATTCAGTGAATCATCGAATCTTTGAACGCACCTTGCGCTCCTTGGTATTCCGAGGAGCATGCCTGTTTGAGTGTCATTAAATTCTCAATCTTTACAACTTTGTGTTGTTCAGATTGGATTTGGGGGTTTTACTGTGAGGAATTCAATTCCTTCACTCCTCTTAAATGCATTAGCTGGAATGCCTCTGCATGTCTGACTATCAGTGTGATAATTATCTATACTGTAGTTGTTGCATGAGGGTATCTGCTTCCTCAATCGTCGCAAGACAAATGACCATCTTGACCTCAAATCAGGTAGRACYACYYGCTGAACTTAAGCATATCAAAA

> LAH38251_Conocybe_safariensis

CATTACAGAGTAAACTTGGTGGGTTGCTGCTGGCTCTCAATTGAGCATGTGCACGCCTGTCATCTTTATCTTTCCACCATGTGCACTTTTTGTAGGTCTGGAATAGACATAAGCCTGGCCACCTGTGGGCTGCTGGAAAGTTGCTGGTTATATCAGCTCTTTCTGTTACTGGCTGTCTTTTCTAGGCTCTATGATTTATCCATATACACCATGTATGTCAATGAACGCAATCATGGGCCCACAAAGCCTATAAACCTATAATACAACTTTCAGCAACGGATCTCTTGGCTCTCGCATCGATGAAGAACGCAGCGAAATGCGATAAGTAATGTGAATTGCAGAATTCAGTGAATCATCGAATCTTTGAACGCACCTTGCGCTCCTTGGTATTCCGAGGAGCATGCCTGTTTGAGTGTCATTAAATTCTCAATCTGTACAACTTTTGTGTTGTCTAGATTGGAAGTGGGGGTTCTTTGTGTGGAATCTAATTCCTTACTCCCCTTAAATGTATTAGCTGGAATGCCTCTGCATATCTGACTATCAGTGTGATAATTATCTATACTGTAGTTGTTGCATGGAGGGTATCTGCTTATCAACCGTCGAAAGACAATTCTATATGACCATCTTGACCT

>LAH38252_Conocybe_safariensis

CATTACAGAGTAAACTTGGTGGGTTGCTGCTGGCTCTCAATTGAGCATGTGCACGCCTGTCATCTTTATCTTTCCACCATGTGCACTTTTTGTAGGTCTGGAATAGACATAAGCCTGGCCACCTGTGGGCTGCTGGAAAGTTGCTGGTTATATCAGCTCTTTCTGTTACTGGCTGTCTTTTCTAGGCTCTATGATTTATCCATATACACCATGTATGTCAATGAACGCAATCATGGGCCCACAAAGCCTATAAACCTATAATACAACTTTCAGCAACGGATCTCTTGGCTCTCGCATCGATGAAGAACGCAGCGAAATGCGATAAGTAATGTGAATTGCAGAATTCAGTGAATCATCGAATCTTTGAACGCACCTTGCGCTCCTTGGTATTCCGAGGAGCATGCCTGTTTGAGTGTCATTAAATTCTCAATCTGTACAACTTTTGTGTTGTCTAGATTGGAAGTGGGGGTTCTTTGTGTGGAATCTAATTCCTTACTCCCCTTAAATGTATTAGCTGGAATGCCTCTGCATATCTGACTATCAGTGTGATAATTATCTATACTGTAGTTGTTGCATGGAGGGTATCTGCTTATCAACCGTCGAAAGACAATTCTATATGACCATCTTGACCT

> LAH38253_Conocybe_safariensis

CATTACAGAGTAAACTTGGTGGGTTGCTGCTGGCTCTCAATTGAGCATGTGCACGCCTGTCATCTTTATCTTTCCACCATGTGCACTTTTTGTAGGTCTGGAATAGACATAAGCCTGGCCACCTGTGGGCTGCTGGAAAGTTGCTGGTTATATCAGCTCTTTCTGTTACTGGCTGTCTTTTCTAGGCTCTATGATTTATCCATATACACCATGTATGTCAATGAACGCAATCATGGGCCCACAAAGCCTATAAACCTATAATACAACTTTCAGCAACGGATCTCTTGGCTCTCGCATCGATGAAGAACGCAGCGAAATGCGATAAGTAATGTGAATTGCAGAATTCAGTGAATCATCGAATCTTTGAACGCACCTTGCGCTCCTTGGTATTCCGAGGAGCATGCCTGTTTGAGTGTCATTAAATTCTCAATCTGTACAACTTTTGTGTTGTCTAGATTGGAAGTGGGGGTTCTTTGTGTGGAATCTAATTCCTTACTCCCCTTAAATGTATTAGCTGGAATGCCTCTGCATATCTGACTATCAGTGTGATAATTATCTATACTGTAGTTGTTGCATGGAGGGTATCTGCTTATCAACCGTCGAAAGACAATTCTATATGACCATCTTGACCT
